# Fructose malabsorption

**DOI:** 10.1186/s40348-016-0035-9

**Published:** 2016-02-16

**Authors:** Karolin Ebert, Heiko Witt

**Affiliations:** Pädiatrische Ernährungsmedizin, Klinikum rechts der Isar (MRI), Technische Universität München (TUM), Gregor-MendelStr. 2, 85354 Freising, Germany; Else Kröner-Fresenius-Zentrum für Ernährungsmedizin (EKFZ), Technische Universität München (TUM), Gregor-MendelStr. 2, 85354 Freising, Germany; Zentralinstitut für Ernährungs- und Lebensmittelforschung (ZIEL), Technische Universität München (TUM), Gregor-MendelStr. 2, 85354 Freising, Germany

**Keywords:** Fructose malabsorption, Glucose transporters, GLUTs, Hydrogen breath test

## Abstract

Incomplete intestinal absorption of fructose might lead to abdominal complaints such as pain, flatulence and diarrhoea. Whether defect fructose transporters such as GLUT5 or GLUT2 are involved in the pathogenesis of fructose malabsorption is a matter of debate. The hydrogen production by colonic bacteria is used for diagnosis with the hydrogen breath test. However, the appropriate fructose test dose for correct diagnosis is unclear. Subjects with fructose malabsorption show increased breath hydrogen levels and abdominal symptoms after fructose administration but do not report any symptoms when fructose is given together with glucose. This beneficial effect of glucose, however, cannot be explained yet but might be used for clinical care of these subjects.

## Background

**Fig. 1:**
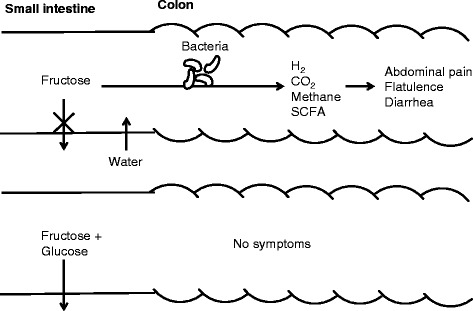
Symptom generation in fructose malabsorption. As consequence of impaired absorption, fructose reaches the colon and is metabolized by colonic bacteria. The products hydrogen, carbon dioxide, methane and short-chain fatty acids (SCFA) and the osmotic water influx cause the typical symptoms. The additional consumption of equimolar amounts of glucose enhances fructose absorption and prevents symptoms

The complex process of sugar digestion and absorption can be disturbed due to defect digestion enzymes or monosaccharide transporters. Lactose malabsorption is caused by variants in the promoter region of the *LCT* gene coding for lactase-phlorizin hydrolase. Consequently, the cleavage of lactose in its components glucose and galactose is impaired [1]. In patients with glucose-galactose malabsorption, mutations in the *SLC5A1* coding region result in truncated or mistargeted glucose-galactose transporter SGLT1. Due to impaired absorption of glucose and galactose, newborns develop life-threatening diarrhoea and dehydration [2]. The molecular and the genetic causes of fructose malabsorption are unknown. Like other sugar malabsorptions, the disease is characterized by colicky abdominal pain, flatulence and diarrhoea. These symptoms are caused by bacterial fermentation of unabsorbed fructose in the colon resulting in production of hydrogen, carbon dioxide, methane and short-chain fatty acids. Moreover, the increased sugar load in the colon might cause osmotic diarrhoea (Fig. 1). Patients should avoid fructose-rich food such as apples, pears and red peppers. Nutrition anamnesis is often not helpful for making diagnosis since affected subjects instinctively avoid fructose-rich foods. For therapy, the beneficial effect of glucose on fructose absorption is used. Patients tolerate fruits such as bananas or grapefruits which contain fructose and glucose in at least equimolar concentrations. Apples are not tolerated because of their high fructose (7 g/apple) and lower glucose (2.3 g/apple) content (ratio 3:1). Furthermore, the simultaneous intake of glucose can avoid symptoms after fructose consumption.

### Are sugar transporter defects responsible for fructose malabsorption?

Since GLUT5 represents the main intestinal fructose transporter, its involvement in the pathophysiology of fructose malabsorption seems obvious. *Glut5* knock-out mice demonstrated decreased fructose uptake in the jejunum and reduced plasma fructose levels compared to their littermates. However, intestinal fructose transport is not completely abolished indicating the presence of other intestinal fructose transporters. The feeding of a diet containing 60 % fructose for 14 days resulted in increased GLUT5 expression in mice [3]. Whether fructose absorption can be enhanced by increased GLUT5 expression due to fructose-rich diet in humans is not investigated so far.

The *GLUT5* coding region was analyzed using SSCP (single-strand conformational polymorphism) in 8 children with fructose malabsorption, 6 healthy controls and 13 healthy parents of patients [4]. The patients were diagnosed by hydrogen breath testing with 1 g/kg fructose and occurrence of symptoms during the test. No *GLUT5* variant was found that correlated with the occurrence of fructose malabsorption. Nevertheless, an involvement of GLUT5 in the pathophysiology of fructose malabsorption cannot be excluded since this cohort was quite small, the age of patients was very young (ranging from 8 months to 5 years, median 2.75 years) and half of the patients showed positive sucrose breath test questioning the diagnosis of isolated fructose malabsorption [4]. Moreover, SSCP analysis has a low sensitivity compared to DNA sequencing, and non-coding regions were not investigated in this study.

**Fig. 2:**
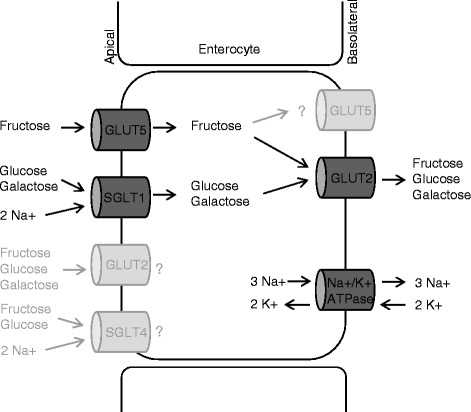
Intestinal sugar transport. Fructose is transported into the enterocyte by GLUT5 and glucose and galactose by SGLT1. Exit of fructose, glucose and galactose is provided by GLUT2. The presence and relevance of GLUT2 and SGLT4 in the apical membrane and GLUT5 in the basolateral membrane are under debate. Verified transport routes are illustrated in *dark grey*, hypothetical in *light grey*

Other fructose transporters such as GLUT2 or SGLT4 are also present in the small intestine and might play a pathogenic role in this condition (Fig. 2) [5–7]. GLUT2 is expressed in the basolateral membrane but the presence in the apical membrane is questionable [8, 9]. If GLUT2 is responsible for the exit of fructose only, an important role for fructose malabsorption is unlikely. *GLUT2* mutations are associated to Fanconi-Bickel syndrome, an autosomal recessive inherited disorder characterized by hepatorenal glycogen accumulation, renal tubular dysfunction and glucose/galactose intolerance. However, no disturbed fructose absorption has been reported in this disease [10].

The hypothesis that intestinal GLUT5 or GLUT2 expression is reduced in fructose malabsorption was tested by Wilder-Smith et al. in 11 adults with fructose malabsorption (positive breath test after ingestion of 35 g fructose in combination with abdominal symptoms) and 15 control subjects. The messenger RNA (mRNA) content and protein expression of GLUT5 and GLUT2 was similar in both groups [11].

Unlike GLUT5 and GLUT2, SGLT4 provides Na^+^-dependent secondary active transport of glucose and possibly fructose. Fructose transport by SGLT4 was not directly shown but an inhibition of glucose uptake by fructose was demonstrated. So far, the expression of SGLT4 in the apical membrane and its role for intestinal fructose absorption has not been investigated [6].

### Is the hydrogen breath test appropriate for diagnosis?

Fructose malabsorption is usually diagnosed by hydrogen breath test (HBT). Since the absorption capacity for fructose is limited in healthy people also, the appropriate fructose dose for this test is under discussion. After ingestion of 50 g fructose, 58 % of 102 subjects demonstrated incomplete absorption with a rise in breath hydrogen over the arbitrarily defined threshold level of 20 ppm. In these subjects, 25 % reported abdominal symptoms during the test period. Twenty-one persons with a positive breath test after ingestion of 50 g fructose repeated the procedure with 25 g fructose. With this lower dose, only 11 % showed incomplete absorption. Moreover, of the 11 % who had abdominal symptoms, only one person showed an elevated breath hydrogen level [12]. Rao and co-workers conducted a double-blind study with 20 healthy subjects: all subjects exhibited negative HBT with 15 g fructose. Two subjects (10 %) showed incomplete absorption with a dose of 25 g fructose but did not report symptoms. After administration of 50 g fructose, 80 % of subjects showed breath hydrogen rise over baseline and 55 % demonstrated abdominal symptoms [13]. When 31 children received 2 g fructose per kg bodyweight (max. 50 g), 71 % showed positive breath test and 13 % had abdominal symptoms [14]. As shown by these and other studies, a dose of 50 g (corresponds to approximately seven medium-sized apples) in adults or 2 g/kg in children exceeds in the majority the intestinal absorption capacity whereas the administration of ≤25 g fructose in adults or ≤1 g/kg in children is thought to be appropriate for diagnosis of fructose malabsorption. Until the reason for fructose malabsorption is unclear, it is hardly possible to define the right dose for HBT and to discriminate between normal and abnormal. Therefore, it is difficult to estimate the prevalence in the general population. A detailed review about hydrogen breath testing in fructose malabsorption was published by Jones et al. [15].

### Age dependence of fructose absorption

There is evidence that the prevalence of fructose malabsorption correlates with the age. After administration of 1 g/kg fructose, 16 out of 23 children aged 1–3 years showed elevated peak hydrogen excretion, whereas only 7 out of 26 older children (4–6 years) demonstrated increased breath hydrogen levels. Furthermore, peak hydrogen was higher in the younger age group [16]. A clear correlation between age and HBT (0.5 g/kg, max. 10 g fructose) was found in a study population of 1093 children and adults, where fructose absorption capacity was rising up to the age of 10 years. From the age of 10 to 79, no differences were observed regarding absorption capacity [17]. On the contrary, the prevalence of lactose intolerance is low in infants and rises with the age. In pediatric populations from Finland and Somalia, the lactase activity decreased mainly between 5 and 10 years of age [18].

### The beneficial effect of glucose

The simultaneous ingestion of glucose can improve fructose absorption and prevent symptoms. In 1984, it was described that children showed increased breath hydrogen excretion after administration of 2 g/kg (max. 50 g) fructose but not after ingestion of fructose together with equimolar amounts of glucose or galactose. Only the three children with the highest breath hydrogen peaks demonstrated abdominal symptoms [14]. This effect was confirmed by Rumessen et al. in 10 healthy adults. An elevated breath hydrogen excretion after ingestion of 50 g fructose was observed in 7 out of 10 people. In contrast, breath hydrogen peaks were normal after simultaneous ingestion of equimolar amounts glucose [19]. Patients with fructose malabsorption can additionally consume glucose if they want to eat fructose-rich food to avoid symptoms. The reason for this absorption enhancing effect is under discussion. Fructose could be passively absorbed due to glucose-stimulated water absorption. The observation that this beneficial effect was not only mediated by sugars but also by amino acids such as alanine supports this hypothesis [20]. However, fructose transport was not enhanced after ingestion of fructose and urate in equal amounts [14]. Another explanation is the presence of a glucose dependent fructose co-transport system [19]. An involvement of the fructose and glucose transporter GLUT2 has been proposed because trafficking of GLUT2 to the apical membrane after glucose load was observed in rats [8]. In contrast, no evidence for GLUT2 presence in the apical brush-border membrane in response to glucose was shown by Röder et al. indicating the involvement of other transporters [9]. In a cohort of six healthy subjects, it was observed that glucose and galactose can prolongate gastric emptying [21]. Possibly, the delayed arrival of fructose in the small intestine due to delayed gastric emptying could explain the increased fructose absorption by simultaneous glucose administration. Moreover, an enhancing effect of glucose and lipids on sorbitol absorption was demonstrated which might be explained by similar mechanisms [22].

### Fructose malabsorption as cause for recurrent abdominal pain

Gijsbers et al. conducted a double-blinded placebo-controlled (DBPC) trial in children with recurrent abdominal pain. First, 121 children performed a HBT with 2 g/kg fructose (max. 50 g). From those children who were positive (65 %), 49 were set on a fructose elimination diet. Sixty-five percent of them reported absence of symptoms during the diet but only 13 out of 31 children (41 %) responded to an open provocation with fructose. Finally, all 8 children which demonstrated symptoms after provocation, showed no symptoms after double-blinded placebo-controlled re-provocation. In summary, none of the children demonstrated positive hydrogen breath test together with the absence of symptoms during elimination and symptoms during the DBPC trial. An explanation why the children showed positive hydrogen breath test after fructose ingestion but no symptoms during DBPC with fructose could be the different dose. During the breath test, a maximum of 50 g fructose (2 g/kg) was given whereas in the DBPC trial, 25 g was consumed over the whole day. However, factors such as the gut microbiota, transit time and diet composition might also cause varying fructose tolerance [23]. As mentioned above, 2 g/kg fructose cannot be completely absorbed in the majority of children. Therefore, the dose is too high to discriminate between fructose absorption and malabsorption. Since the elimination and provocation was conducted at home, it is possible that children did not strictly follow the elimination and provocation guidelines leading to inconsistent results. Nevertheless, fructose malabsorption was most likely not the prominent factor for abdominal pain in this cohort.

### Dietary fructose restriction in IBS

High-fructose consumption can trigger symptoms in patients with irritable bowel syndrome (IBS). With a dose of 25 g fructose, 22 % (20/90) of IBS patients showed positive hydrogen breath test. Amongst fructose malabsorbers, 35 % had abdominal symptoms during the test whereas 26 % of the fructose absorbers demonstrated symptoms [24]. With the same fructose dose, 10 % (2/20) of healthy adults demonstrated elevated breath hydrogen without any symptoms [13]. Fructose intake is restricted within the low FODMAP (fermentable oligo-, di- and monosaccharides and polyols) diet often recommended for IBS patients. In a cohort of 90 IBS patients, symptoms improved during this diet. Moreover, the occurrence of fructose malabsorption was associated with improvement of symptoms [25]. A further study in 67 IBS patients showed symptom amelioration after standard IBS diet and low FODMAP diet without significant differences between the two diets [26]. However, potential undesirable side-effects of long-term restricted diets should be taken into account.

## Conclusion

The pathophysiology of fructose malabsorption remains unclear. If there are genetic or epigenetic variations in intestinal fructose transporters such as *GLUT5*, *GLUT2* or *SGLT4* needs to be elucidated. Genetic variants within the *GLUT5* coding regions are unlikely but not excluded as reason for impaired fructose absorption. There are first hints that GLUT5 and GLUT2 expression is not lower in affected subjects. Probably, there are different mechanisms leading to this condition. To elucidate the underlying mechanism, genetic analysis in well-defined cases and controls should be performed either by candidate gene approach or complete genome sequencing. Because the absorption capacity for fructose is highly individual, it is difficult to determine the right dose for HBT. There is agreement that 50 g fructose in adults or 2 g/kg fructose in children exceed the absorption capacity of the majority. A dose of ≤25 g in adults or ≤1 g/kg in children seems to be appropriate. The variance of breath test results at different times of the day and the reproducibility should be assessed. It remains unclear why increased gas production in the intestine during HBT does not necessarily cause abdominal symptoms. Possible, the intestinal sensitivity and pain perception is highly individual. Whether fructose malabsorption is more common in IBS patients needs to be verified. However, restriction of fructose and other fermentable saccharides can be helpful in therapy. To clarify the absorption enhancing effect of glucose, it is important to understand the mechanisms of intestinal sugar transport and to gain insights into possible interactions of sugar transporters. To address this question, future studies should verify the assumption that different amino acids also promote fructose absorption.

## Abbreviations

DBPC: double-blinded placebo-controlled
GLUT: glucose transporter
HBT: hydrogen breath test
SSCP: single-strand conformational polymorphism
